# Supplementary material for: Are carbon emissions trading and green financial instruments synergistic? -Comprehensive quantitative research based on content analysis
Source: PLoS One. 2024 Mar 7;19(3):e0298601. doi: 10.1371/journal.pone.0298601 (PMC10919601; doi:10.1371/journal.pone.0298601)
Supplement: S1 Data — (ZIP) [file pone.0298601.s001.zip › Data/地级市Z_F工作报告.zip!/║■▒▒/╦μ╓▌╩╨/└┤╫╘╣1⁄2╓┌║┼í╛┬φ┐╦╩2╛▌═°í┐_2012.docx]

各位代表：

现在，我代表市人民政府向大会报告政府工作，请予审议，并请市政协委员和各位列席人员提出意见。

一、过去五年工作回顾

过去五年，是极不平凡的五年，是打基础、管长远、经济社会突破性发展的五年。五年来，面对国际金融危机、特大旱涝灾害和日益趋紧的宏观形势，我们坚持以科学发展观为统领，深入贯彻落实党中央国务院和省委省政府决策部署，团结依靠全市人民，推进工业兴市，统筹城乡发展，着力改善民生，圆满完成了市二届人大确定的各项目标任务，实现了“十一五”规划的顺利收官和“十二五”规划的良好开局。

——国民经济快速增长。五年来，全市主要经济指标持续保持超过计划预期、高于全国全省平均水平的“一超两高”发展态势，呈现出发展提速、总量倍增的明显特点：预计2011年实现地区生产总值465亿元，年均增长14.1%，是2006年的1.9倍；固定资产投资370亿元，年均增长39%，是2006年的5.2倍；外贸出口8.87亿美元，年均增长41.1%，是2006年的5.6倍；规模工业增加值190亿元，年均增长23.7%，是2006年的2.9倍；地方财政一般预算收入17.07亿元，年均增长29.1%，是2006年的3.6倍；社会消费品零售总额234亿元，年均增长17.7%，是2006年的2.3倍；城镇居民人均可支配收入17400元，年均增长16.3%，是2006年的2.1倍；农民人均纯收入7400元，年均增长15.6%，是2006年的2.1倍，基本与经济发展相同步。

——经济结构趋于优化。工业经济突破性发展。预计2011年，全市实现规模工业总产值680亿元，五年年均增长31.8%，是2006年的4倍；规模以上工业企业达到447家，其中产值过亿元企业200家，规模企业税收贡献额在全省市州中处于先进行列。在省委省政府的大力支持下，成功创建了中国专用汽车之都，并提升为部省共建战略；专用汽车及零部件、香菇加工、风机制造跻身全省重点产业集群，汽车机械、农产品加工产值双双突破200亿元大关，电子信息、光伏、新材料等战略性新兴产业快速成长。齐星集团进入全国机械行业500强和全省100强，“齐星”、“大力”、“程力威”被认定为中国驰名商标。农业基础更加稳固。大兴农田水利，抗灾能力进一步增强。五年间，我们先后成功抗击了2009年“7.17”特大洪水和去年60年一遇的特大干旱等自然灾害，粮食连续8年增产、农民连续12年增收。大力发展特色农业，农业规模化和产业化步伐进一步加快，建成了全国最大的食用菌出口基地，农产品出口总量居全省首位；建成了6个国家级农业标准化示范区，同星农业、裕国菇业成为国家级农业产业化龙头企业，大洪山牌香菇、集蜂堂牌蜂蜜荣获全省十大名牌农产品称号；广水市成为全国生猪调出大县、旱涝保收高标准农田示范县和农村饮水安全示范市，随县成为全国产粮大县、生猪调出大县和全省“三农”发展先进县，曾都区成为全省农产品加工“四个一批”、农田水利建设先进县（市、区）。文化旅游成果丰硕。世界华人炎帝故里寻根节连续三年成功举办，已经成为全省重要的文化节会品牌；中国音乐家协会授予随州“中国编钟之乡”称号，炎帝、编钟这两张文化名片已叫响全国。叶家山墓地的文物发掘与保护被列为2011年中国十大考古发现之首；曾随文化研究在国内外产生深刻影响。炎帝故里、玉龙温泉跻身国家4A级景区，这些都极大地促进了旅游业的突破性发展。五年累计接待游客2610万人次、年均增长24.4%，旅游总收入142亿元、年均增长36.1%。

—— 三大需求繁荣活跃。投资需求持续拉高。全社会固定资产投资五年累计完成1119亿元，实现了两年翻一番。开发区建设成为拉动投资增长的重要力量，五年共建成4个省级开发区，市开发区在全省省级开发区综合考评中居第五位；一批产业项目、基础设施项目加快推进，武康铁路增建二线、随岳高速和麻竹高速随州段建成通车，全市境内高速公路通车里程达到203.4公里，初步构筑起以交通促流通的大交通格局。社会消费日趋旺盛。传统服务业提档升级，现代服务业加快发展，浙江银泰、中商百货、武商集团等一批大型商业企业进驻随州，消费多样化需求日趋增强。外贸出口逆势增长。大力扶持出口主体，扩大对外贸易，建成国家外贸转型升级示范基地，培育出口企业300家、五年净增140家。出口产品呈现出多元化格局，覆盖139个国家和地区。即使是2008年金融危机以来，我市对外贸易仍然保持了逆势攀升势头，被省政府授予全省扩大开放先进单位。

—— 城乡面貌显著变化。坚持规划先行，城市建设大踏步推进。以启动新一轮城市规划为契机，以新火车站建成通车及“九路一桥”配套完成为标志，拉开了城东、城南新区框架，建成区面积扩大了20平方公里。五年累计投资126亿元，先后建成了白云湖水库、滨湖风光带、滨湖体育公园、神农公园、波导大桥、波导大道、白云大道、迎宾大道、炎帝大道、编钟大道、神农大道，新建主干道51公里，改建道路20.7公里，整治背街小巷219条；加快建成了市博物馆、市图书馆、市民中心等一批文化体育设施；相继实施了城市燃气、垃圾处理场、污水处理厂、内河治理、明珠广场和白云山森林公园改造等一批重大市政工程。城市绿化、美化、亮化水平进一步提升，新增绿化面积360万平方米，“一河两岸”的规划建设荣获国家“人居环境范例奖”。坚持精细化管理，城市品位加快提升。健全城市管理长效机制，着力构建市区一体、分工明确的“大城管”格局，严厉查处违法建设，持续开展“三城同创”，我市被授予省级文明城市、卫生城市、园林城市、森林城市和“楚天杯”优胜城市，在2011年全省城市建设管理两轮考核评比中分别荣获第四名和第三名的优异成绩。坚持以特取胜，县域经济竞相发展。完成新随县和曾都区的机构组建，随县新县城建设顺利启动并夺取2010年度全省县域经济考评进位奖，曾都区连续五年被评为全省县域经济考评先进单位，广水市自去年以来已呈现出加快发展的良好势头，市开发区、曾都区和大洪山城乡一体化发展取得新进展。大力推进随南和广水北三镇试验区建设，建成 115个居民点。通村公路达到4100多公里，提前两年实现村村通水泥路的目标。投资8.36亿元，实施了53座大中型和重点小型水库除险加固，新建和改造当家大堰塘5300口；兴建各类供水工程1780处，解决了77.9万农村人口的饮水安全问题。完成土地整理38.8万亩，新增耕地2.4万亩；投入2.2亿元，完成农业综合开发面积20万亩。实施“百镇千村”示范工程，新建沼气池和改厨、改圈、改厕“一建三改”8万多个。农村环境面貌不断改善，应办、广办进入全省百强，郝店成为全省新农村建设试点镇，洪山镇、武胜关镇、吉祥寺村等一批镇村先后跻身于省级特色镇、重点中心镇、生态镇和生态村的行列。

—— 人民群众得到实惠。社会保障不断完善，各项事业长足进步，和谐随州成效显著。城镇五年累计新增就业近10万人，失地农民养老保险在全省率先实行，五项保险待遇逐年提高并惠及百万居民，新农合和城镇医保基本覆盖，城乡居民养老保险加快推进，城乡低保应保尽保。累计投入10亿元，改造校舍面积15万平方米；争取资金近8亿元，资助农村贫困生19.7万人次。收回了随州职院的举办权，建组了湖北现代教育集团。重大疾病防治措施有力，突发公共卫生事件得到及时妥善处置。建成保障性住房1.4万多套，发放廉租补贴9000多户，南水北调移民安置任务在全省率先完成。扶贫开发力度加大，农村贫困人口减少近9万人。五年累计实施60件民生工程和实事项目，住房难、上学难、看病难、出行难等涉及人民群众切身利益的问题得到较大程度地缓解。

—— 精神文明建设、民主法治建设和政府自身建设全面加强。文明社区、文明镇村和行业“十佳”等精神文明创建活动扎实开展，公民思想道德素质和社会文明程度明显提高，“光满服务队”成为全国精神文明建设的一面旗帜。依法治市进程加快，“五五”普法顺利完成，“六五”普法启动实施，“有话就说”纠纷调处机制成为全省平安创建一大品牌，广水市、随县荣获全省平安县（市、区）称号，我市创建为全省双拥模范城，广水市蝉联全国双拥模范城。大力推进以责任为核心的法治政府和服务型政府建设，坚持执行重大事项向市人大及其常委会报告、向市政协通报制度，自觉接受各方面的监督，五年共办理人大议案、建议和政协提案819件，办理质量不断提高。完成市政府机构改革，深化行政审批制度改革，加强目标管理，推动了各项工作的落实。反腐倡廉取得积极进展，努力做到了以良好的政风带民风。

各位代表！五年的巨大变化，来自于年度目标全面完成的有力支撑。尤其是刚刚过去的2011年，既是我市“十二五”跨越发展的起始之年，也是争先进位取得实质性进展的一年，元至11月，全市工业增加值、财政收入、固定资产投资、社会消费品零售总额等主要经济指标增幅进入全省前五位，谱写了随州跨越赶超的新篇章。

（一）突出工业兴市，总量质量进一步提升。“专汽之都”建设取得新进展。中国机械工业联合会和省政府共同在随州举办中国专用汽车发展论坛，市政府与省汽车行业协会及其骨干企业签署合作协议。汽车行业市场整顿取得成效，新落户厦工楚胜专用车、尚沃改装车、上汽商用车等一批大项目，20公里的专汽走廊初具规模。支柱工业增势强劲。汽车机械、食品加工、纺织服装、建材、化工、光伏电子、生物医药等七大板块实现产值580亿元，同比增长40%。光伏电子、有机硅和光电子材料等新兴产业迅速兴起，多晶硅项目建成投产，太阳能硅片项目启动实施。市场主体加快成长。在重点企业稳步增长、齐星集团销售收入即将跨过50亿元大关的同时，全民创业步伐进一步加快，全年新增市场主体超过1.5万户，日均42户，为历年之最。

（二）立足抗旱夺丰收，农业产业化步伐进一步加快。在60年一遇的四季连旱面前，坚持把抗大旱、保饮水、保三夏、保稳定作为一项政治任务，深入开展“万名干部进万村入万户”活动，积极推广新技术、新模式，有效稳定了农村生产形势。除油料以外，各类农产品普遍丰收，粮食总产161.1万吨，同比增长3.5%。与此同时，大力推进农业产业化经营，新增省级以上龙头企业6家，新发展农民专业合作社429个、总数达到1105个；新建了一批高产创建示范片，马铃薯千亩连片单产创全国最高纪录、最优模式。“亿只鸡”工程顺利推进，省级、国家级标准化养殖小区分别发展到22个和4个，建成木本油料基地11.7万亩、累计达到22万亩。全面落实强农惠农政策，整险加固100座小型水库，新建和改造大堰塘5730口，解决了7万农村人口的饮水安全问题。扶持建设生态家园，新建户用沼气池5300口，绿色家园示范村发展到200多个。完成20个村的扶贫开发任务，1万多贫困人口实现稳定脱贫。

（三）彰显文化特色，第三产业进一步繁荣。充分发挥文化资源优势，着力打造炎帝故里、编钟之乡、佛教名山等文化品牌，辛卯年世界华人炎帝故里寻根节取得圆满成功，重点景区景点开发建设步伐加快，大洪山宝珠峰金顶如期建成开放，炎帝故里入选“灵秀湖北十大旅游名片”、大洪山入选“灵秀湖北十大旅游新秀”，随县成为湖北省旅游强县。城乡商贸活跃壮大，流通网点发展到2万多个，“农家店”覆盖90%的行政村，“新网工程”加快推进；销售家电下乡产品16.5万台，发放补贴4413万元。食品、药品、成品油、农资等专业市场监管进一步加强，质量安全得到保证。

（四）实施项目拉动，发展后劲进一步增强。各级各类开发区和工业园区基础设施加快完善，市开发区成为省级高新技术产业园，10平方公里的城东工业园实现七通一平，深圳工业园如期开园。全市累计实施500万元以上投资项目1273个、同比增加300个，全社会固定资产投资同比增长35%，其中实际利用内联资金75亿元、同比增长30%。齐星集团10万辆车身改造、厦工楚胜、全力机械、三环铸造、奥龙专用车、双剑高速风机等一批过亿元工业项目顺利推进。

（五）坚持建管结合，城镇形象进一步改善。投资近20亿元，新建了新火车站站前大道、波导大道、清河东路、波导大桥等“九路一桥”基础设施项目，实现了新老城区的循环互通。不断加强城市绿化、亮化、美化工作，“人人参与，洁美随州”行动收到明显成效，城市建设和管理工作名列全省前茅。广水市被评为省级卫生城市，随县厉山老镇改造、行政办公新区建设全面启动。全市当年新创建4个特色镇、3个重点中心镇和17个宜居村庄。

（六）着力保障民生，社会管理进一步加强。多途径解决就业难、招工难的结构性矛盾，城镇新增就业1.6万人，农村劳动力转移就业近2万人。社会保险参保人数和基金规模大幅增加，企业退休人员和被征地农民养老金继续提高。开征价格调节基金，发放临时价格补贴，保障困难群众的基本生活。致力打造“七彩阳光”教育品牌，中考网上阅卷等多项工作经验在全省推广；高中教育质量稳步提升，本科万人比上线率全省第一；中职教育走在全省前列，随州职院顺利通过教育部第二轮评估，随州技师学院成为国家级示范学校。科技创新工作取得成果，我市荣获全国科技进步先进市称号。文化阵地建设进一步加强，新建农民健身工程50多处，农家书屋达到1039家，广播电视“村村通”工程加快推进。医疗卫生服务能力不断提升，基本药物制度改革全面实施，新农合参合率达到97.2%，定点医疗机构即时结报实现“一卡通”。加强社会救助，启用市儿童福利院，新建了4个乡镇中心福利院，免除特困对象殡葬服务费114万元。加快安居工程建设，新增廉租房228套、租赁房3814套、棚户区改造3600户，增发货币化补贴1309户。住房公积金制度加快覆盖，新增归集资金1.8亿元；发放个人贷款1.45亿元，同比增长近4倍。人事、编制、审计、统计、国调、司法、档案、保密和地方志工作有了新进步；宗教、外事、侨务、对台和烟草专卖工作取得新成绩；气象、供水、供电、通讯和无线电管理工作保障有力；红十字会、无偿献血、妇女、儿童、老龄和残疾人等各项事业继续健康发展。全面贯彻落实人口和计划生育政策，出生人口性别比趋于合理。加强国土资源管理，多途径盘活资源存量，缓解用地紧张的矛盾。扎实抓好节能减排，万元GDP能耗和化学需氧量、氨氮等约束性指标全面完成。加强和创新社会管理，不断完善公共服务，认真做好新形势下的群众工作，公众社会治安满意度调查位居全省前列。高度重视安全生产和公共安全，严厉查处各类违法违规案件，安全形势总体稳定。食品药品监督、质量技术监督工作迈上新台阶，民防工作走在全省前列，双拥、国防、民兵预备役工作得到加强；驻随部队积极支持地方建设，在急难险重的工作中发挥了巨大的作用。

在总结成绩的同时，我们必须保持清醒的头脑，正视工作中的不足和发展中的差距：经济总量不大，产业层次和整体水平不高，缺乏大企业、大项目支撑，自主创新能力与核心竞争能力不足；人口资源环境矛盾显现，土地、能源、资金等制约加剧；财力薄弱，公共服务能力与群众期望仍有差距；统筹城乡发展、维护稳定、创新社会管理的任务仍十分繁重；特别是少数部门及其工作人员仍不同程度地存在“庸懒散”的现象，已成为优化环境中的突出问题。对此，我们必须高度重视，下大决心、花大气力，认真地加以解决。

各位代表！回顾历史，展望未来，我们深切感受到，在今后的发展道路上，必须始终做到“四个坚持”：即坚持解放思想、扩大开放，用解放思想的成果去破解发展中的难题，用扩大开放的力量去激发促进跨越的活力；坚持科学发展、奋力赶超，用科学发展的理念去统筹城乡的协调共进，用奋力赶超的业绩去积累可持续发展的后劲；坚持工业兴市、“两化”并举，用工业兴市的旗帜去凝聚全市上下的发展共识，用城乡面貌的改善去增强区域经济的竞争力；坚持文化引领、管理创新，用文化资源的优势去积累发展的软实力，用社会管理创新的举措去提升人民群众的幸福感。

各位代表！建市十年以来，我们先后走过了艰辛创业的五年、突破发展的五年，正在迈向跨越发展的五年。2011年及过去五年的发展成就，是省委省政府和市委统揽全局、科学决策的结果，是市人大、市政协加强监督、大力支持的结果，是历届政府励精图治、不懈奋斗的结果，更是全市人民和衷共济、团结拼搏的结果。在此，我代表市人民政府，向各位人大代表、政协委员，向全市各条战线上辛勤工作的广大干部群众，向驻随部队、武警官兵和政法干警，向各民主党派、工商联、人民团体和驻随单位，向所有关心支持随州发展的老领导、同志们、朋友们致以崇高的敬意和衷心的感谢！

二、今后五年的政府工作

今后五年，是我市推动经济大跨越、文化大繁荣、城乡大发展、社会大进步的关键五年。按照市第三次党代会的决策部署，政府工作的总体要求是：坚持以邓小平理论和“三个代表”重要思想为指导，深入贯彻落实科学发展观，高举跨越式发展的大旗，强力推进工业兴市主战略，着力提高发展速度，做大经济总量；着力调整优化结构，转变发展方式；着力实施项目建设，增强发展后劲；着力统筹城乡发展，深化改革开放；着力创新社会管理，保障改善民生，加快建设中国专用汽车之都、湖北最大的农产品出口基地、全省重要的光伏产业基地、世界华人谒祖圣地和中国优秀旅游目的地，加快推进工业强市、农业强市、文化旅游强市建设，奋力推动随州由中等城市向区域性大城市的跨越。

经济社会发展的主要奋斗目标是：确保“两个高于”和“六个翻番”，即主要经济指标高于“十一五”增长水平、高于全省平均水平，到“十二五”末，地区生产总值、固定资产投资、规模工业增加值、地方财政一般预算收入、社会消费品零售总额和外贸出口实现翻番。其中，地区生产总值年均增长13%以上、突破1000亿元，工业增加值年均增长18%以上，城镇居民人均可支配收入、农民人均纯收入年均分别增长11%和10%，促进各项社会事业全面协调发展。

（一）推动经济大跨越。工业经济要确保突破“双千”目标。坚持不懈地发展大产业、培育大企业，力争到“十二五”末，规模工业总产值达到1500亿元；全力支持成长性好、竞争力强的企业做大做强，力争规模企业突破1000家，其中2家产值过100亿、5家过50亿、20家过10亿，力争3家以上企业成功上市。园区建设要瞄准国家级开发区或高新区的目标。扩大招商引资，加强资源整合，促进集约发展，使市开发区产值达到500亿元以上，成为全市经济发展的重要增长极。农业发展要实现种养加的同步推进。稳定粮食等主要农作物面积，进一步提高产业化水平，发展特色农业，确保农产品加工产值超过400亿元。第三产业要加快转型升级步伐。大力发展社区服务、现代商贸、物流配送、金融保险、信息咨询等生活性、生产性和科技型服务业，加快引进国内外大型商贸流通企业，调整优化服务业布局，建设城东新区中心商务区、农产品批发市场。县域经济要进一步争先进位。全面落实“一主三化”的方针，推动县域经济超常规发展，力争“十二五”末，曾都争创全省十强、广水重返二十强、随县进入全省“第二方阵”。改革开放要纵深推进。深化大部制、行政审批制度、产权制度和事业单位的改革，完善投融资体制机制，增强经济社会发展活力。实施大开放战略，更加注重本土企业与外来大型企业集团的战略性重组，着力培植一批成功嫁接的龙头企业；更加注重招大商、招外商，着力引进一批具有产业优势的外资企业；更加注重总量与质量的统一，着力提升招商引资的科学化、专业化水平。

（二）推动文化大繁荣。全面贯彻落实党的十七届六中全会精神，把提高文化软实力放在富民强市的突出位置，以高度的文化自觉和文化自信，推动文化建设，提高科学化水平。进一步传承历史文化，延续和固化城市记忆，擦亮炎帝文化、编钟文化、佛教文化、红色文化等文化名片，坚持高标准办好世界华人炎帝故里寻根节，积极申报炎帝神农故里作为全国海峡两岸交流基地；策划创作一批文艺作品，深层次提升随州文化品牌的影响力。着力构建全覆盖、普惠型的公共文化设施体系，加快建设文化主题公园、城市规划展览馆、图书馆、体育场、传媒中心等标志性建筑及青少年、老干部活动场所，大力实施广播电视“户户通”和农村电影放映工程；扶持发展文化创意产业，做大做强文化骨干企业，全方位激发文化的经济潜力。加快推进特色地域文化与山水生态旅游的相融共生，使旅游的过程成为游客体验文化、欣赏文化的过程；大力创建一批4A、5A级景区，力争年接待游客突破1200万人次，旅游总收入过百亿元，着力把旅游业培育成战略性支柱产业。

（三）推动城乡大发展。突出规划的前瞻性、针对性和导向性，有序推进市、县、乡、村四级城镇体系建设。率先发展中心城市。加快完善功能设施，拓展新城区，改造老城区，突出山水生态园林特色，使中心城市建成区面积拓展到80平方公里。实施畅通工程，改扩路口，清理新建停车场，改建公交停靠点，切实解决老城区交通拥堵问题。纵深推进城市创建工作，着力创建国家园林城市、森林城市、卫生城市和环保模范城市。加强城市管理，严格规划执法，持续开展“人人参与、洁美随州”行动。协调推进城镇建设。支持随县加快新县城建设，支持广水市拓展城市新区，支持曾都区推进城乡一体化进程。着力改善村庄面貌。突出特色发展，大力培育一批中心镇、特色镇，力争创建3个省级试点镇、4个省级旅游名镇、100个宜居村庄，力争城镇人口3万人以上的建制镇发展到15个。

（四）推动社会大进步。完善社会保障。力争城镇五年累计新增就业10万人以上，农村劳动力转移就业20万人，城镇登记失业率控制在5%以内；逐步提高企业职工最低工资标准。基本保障农村饮水安全，每年解决1万农村人口的稳定脱贫问题，特困人口实现应保尽保，农村绝对贫困现象基本消除。全面实施新农保制度，五项社会保险扩面新增10万人次。加快安居工程建设，缓解城乡低收入群体的住房难问题。深化医药卫生体制改革，健全完善覆盖城乡的公共卫生服务、医疗服务、医疗保障和药品供应体系，基本适应人民群众多层次的医疗卫生需求。坚持科教兴市。加大科教投入，深化产学研合作，不断提高科技的支撑力；优化中小学校布局，均衡发展义务教育，规范发展学前教育，加快发展职业教育，提高高中教育管理水平。创新社会管理。坚持以人为本、重心下移，突出社区建设，完善服务机制，构建和谐社区。加强人口和计划生育工作，综合整治出生人口性别比，优化计生服务，提高人口素质。加强国土资源监管和开发利用，严守耕地红线。加强环境保护，着力节能减排，促进可持续发展。扎实开展安全生产整治行动，加大消防工作力度，杜绝发生重特大事故。认真做好信访维稳工作，狠抓社会治安综合治理，严厉打击各类违法犯罪，全力争创全省平安市、优秀平安县（市、区）和全国双拥模范城。

三、2012年的重点工作

2012年是全面贯彻落实市第三次党代会精神的重要一年，是实现本届政府五年任期目标的第一年，必须开好头，起好步，力争全市地区生产总值增长12%以上，规模工业增加值增长18%以上，固定资产投资增长26%以上，地方财政一般预算收入增长15%、外贸出口增长10%，实现内联资金、实际利用外资分别增长20%和8%，社会消费品零售总额增长17%，城镇居民人均可支配收入和农民人均纯收入均增长11%，居民消费价格涨幅控制在4%左右，单位生产总值能耗下降3%，二氧化碳、二氧化硫、化学需氧量、氨氮和氮氧化物“五项约束性指标”全面完成省定目标。

上述目标是一个预期目标，是综合考虑中央确定的2012年经济工作“稳中求进”的总基调和我市有利条件及制约因素，兼顾当前和长远、需要和可能而提出来的。保持这样一个相当的增长速度，有利于形成稳中求进的发展态势，“稳”是为了更好更快地“进”，既要在发展速度上求进，也要在发展质量、发展动力、发展环境上求进，以激发全市上下用更高的奋斗目标确保超额完成计划预期的发展信心。重点抓好六个方面的工作：

（一）做大做实做强工业经济

着力提升优势产业的支撑力。围绕实现专用汽车千亿元产业的发展目标，深化部省合作战略，提高中国专用汽车发展论坛对外来企业的吸引力，增强其对产业发展的带动力，以齐星集团、恒天汽车、厦工楚胜、中国重汽、航天双龙、三环铸造、全力机械等企业为引领，扩大整车生产规模，加快研发关键零部件，构建企业密切协作、产业集群发展、比较效益突出的良性发展格局，力争当年实现汽车机械总产值280亿元，进一步增强中国专用汽车之都的综合实力；围绕创建中国风机名城，以湖北风机厂、双剑风机、中意机电等企业为重点，实施扩能改造，引进上下游企业；围绕构建多点支撑、竞相发展的产业格局，进一步加快医药、化工、建材、纺织服装等产业的升级步伐，增强支柱地位；围绕打造全省最大的农产品生产加工出口基地，以同星农业、裕国菇业、三友食品、神农生态等企业为龙头，加快开发高档次、高效益、多品种、系列化的特色农产品，提高精深加工水平。

着力培植新兴产业的竞争力。加大新能源、新材料、电子信息、生物医药等新兴产业领域的招商引资力度，加快推进华润风电项目，积极支持多晶硅项目扩能改造和战略重组，延伸光伏产业链；大力推进有机硅新材料的研发和产业化。引导泰晶科技、美亚迪电子等企业扩大规模，以深圳工业园为载体，加快推动通讯电子、集成电路、LED、机电一体化等企业的落户进程。

着力增强市场主体的拉动力。继续实施“市场主体增量行动”，做多增量、做活存量、提升质量、增强能量，扶持全民创业，力争当年新增市场主体1.5万户以上；继续抓好“中小企业成长工程”，力争新增规模以上工业企业50家，着力培育一批外向型经济增长点；落实好国家支持小微企业的各项政策措施，加强中小企业服务体系建设。

着力提高生产要素的保障能力。积极推进银企合作，努力增加对重点项目、优势产业、中小企业、县域经济、“三农”等领域的信贷投入，力争当年新增贷款增幅位居全省前列。积极推动直接融资，充分运用企业上市、产业投资基金、创业投资、风险投资等多种方式融通资金。创新用地保障机制，提高节约集约用地水平，推进迁村腾地、土地增减挂钩等节约用地模式。积极实施劳动力继续培训工程，切实解决好就业难、用工难的突出矛盾。

（二）做大做实做强特色农业

继续巩固农业基础设施的保障功能。大规模开展农田水利建设，新建泵站39座，新增和改善灌溉面积7.6万亩；深入开展“万名干部进万村挖万塘”活动，通过以奖代补、驻点帮扶和“一事一议”等方式，大力实施“千村万塘”工程；投资4300万元，完成大型灌区续建配套与节水改造年度任务；继续抓好病险水库除险加固、农村饮水等工程建设。有序推进土地整理和复垦开发，确保基本农田面积不减、质量提高。加强农村能源建设，完成“一建三改”4000户。

加快提高特色农业的规模和效益。高度重视粮食生产，建好10个万亩粮棉油高产示范片、10个果茶菜标准示范园，稳定提高粮食等主要农产品的产量和品质。大力实施“四个一批”工程，加快中南地区最大的肉鸡生产基地建设，抓好畜牧大镇和畜牧小区建设，继续推进食用菌、干鲜果、畜禽等农产品深加工。加快新一轮菜篮子工程建设，落实最低保有量，建成充足的城市保障性蔬菜基地，新建15家平价菜店。积极探索生态农业、休闲农业、循环农业发展的新模式。加强林业资源保护，开展低产林改造，鼓励发展木本油料、苗木花卉、森林旅游等产业，加快建设林业科技产业园。

完善“三农”工作体系，全面推进新农村建设。统筹推进随南和广水北三镇试验区建设，加快推进农村环境连片整治示范工程。推动土地适度流转，加强农业机械化应用，创建一批标准化示范区，探索农村集体经济发展的新路子。实施扶贫开发“百千万”素质工程，完成20个贫困村的整村推进任务。大力发展务工经济，广泛开展送科技、送项目、送资金等涉农服务，帮助农民科学致富、创业致富。加强农村经纪人队伍、流通组织建设，实施“双百工程”，新增农民专业合作社100个，新建“新网工程”流通网点100个，提高农民组织化程度，进一步畅通大宗农产品销售渠道。深化农村综合改革，严格控制农民负担。

（三）做大做实做强项目投资

进一步推进园区建设，支撑投资增长。支持市开发区加快项目建设，在确保24个在建项目今年全部建成投产的同时，进一步加大招商引资力度，力争引进资金50亿元。支持曾都开发区增强产业发展的承载能力，新增产值150亿元以上；支持随县开发区加快基础设施和项目建设，力争新增利税过亿元；支持广水十里、杨寨等工业园区完善配套、聚集发展。

进一步推进城镇建设，拉动投资增长。树立全域发展理念，创新经营城市思路，多渠道筹措建设资金。主城区按照拉开框架、完善功能、提升形象的要求，重点启动城市规划展览馆、数字化城市管理指挥中心建设和东护城河治理工程；续建季梁公园、滨湖体育场、青年路大桥、望城岗污水处理厂二期、城南垃圾处理场、花溪河治理、编钟大道北端等七大工程；延伸滨湖风光带，配套建设绿化带和小绿地、小游园；完善城市路网，加快建设城东新区七条道路、新建改造老城区四条道路、建成大十字街人行天桥。积极支持随县新县城和广水新区建设，继续实施“楚天杯”、“神农杯”创建和“百镇千村”工程，以随南试验区东线为样板，建设一批文明、靓丽、宜居的集镇和村庄，着力推进镇村一体化。

进一步推进重点项目建设，保障投资增长。完善重大项目建设工作机制，大力实施一批基础产业项目，重点推进厦工楚胜、大力精工、成龙威、腾宇专汽、中航工业、上汽集团、西门电机等一批企业项目建设；鼓励商贸龙头企业新增网点、扩张规模，大力支持粮食、汽车、钢铁物流园建设，新建“农家店”60家；加快实施城乡交通设施项目，推进麻竹高速随州西段建设，加快县乡公路的新建和改造步伐；续建随州一级货运站，支持随县和广水客运站建设。优化配置招商资源，瞄准中国500强和重点行业100强企业，借助中博会等各类经贸洽谈平台，积极推介、引进项目；加强与央企紧密对接，确保签约项目成为实质性投资。

（四）做大做实做强文化旅游

坚持用社会主义核心价值体系引领社会思潮，提升广大市民的文明素质和社会文明程度。按照公益性、基本性、均等性、便利性的要求，加快城乡公共文化服务体系和文化体育设施建设，大力发展广场文化、社区文化、企业文化、村庄文化、校园文化和网络文化。深化文化体制改革，分类实施文艺院团改革，加快公益性文化事业单位改革，完成文化市场执法机构改革，不断解放和发展文化生产力。着眼于促进文化与经济的融合，推动文化产业拓展空间、提档升级，进一步丰富办节思路，精心筹办寻根节等系列活动，提高节会的品牌和效益。加强名城风貌、文物古迹和城市周边山体的保护，更多地融入历史文化元素；积极推进擂鼓墩国家考古遗址公园建设，做好叶家山墓地文物的发掘和保护工作，多角度凸显随州的地域文化特色。加快重点景区景点建设，完成炎帝故里景区农耕园、百草园和养生馆等配套设施，不断丰富景区文化内涵；推动大洪山开发与海航集团的实质性合作，启动慈恩寺景区二期建设，着力塑造“佛教名山、养生天堂”的特色与魅力；加快徐家河、三潭景区综合开发，促进洛阳千年银杏谷、明玉珍故里、桐柏山太白顶等景区提档升级。继续实施旅游经济强县（市、区）和旅游名镇、名村创建活动。

（五）做大做实做强科技教育

坚持把科技创新作为跨越式发展的核心动力，围绕“精深、特色”，打造“随州原产”；围绕“著名、驰名”，叫响“随州品牌”。以企业创新为主体，鼓励企业与高校、科研院所共建研发中心、工程技术中心和重点实验室，力争在专用汽车及零部件、风机制造、医药化工、光伏电子、有机硅新材料、生物质能源、农产品加工等领域培育一批具有较强竞争力的高新技术企业；以完善服务为保障，健全信息、咨询、培训、融资等中介体系，鼓励发展生产力促进中心、科技孵化器等服务机构，加快建设中国专用汽车研究院；以领军人物为骨干，坚持培养、引进、使用并重，建立激励机制，加强个性化服务，让优秀人才有荣誉、有地位、有财富。

始终坚持把教育摆在优先发展的战略地位，加大投入力度，改善薄弱学校办学条件；落实学前教育三年行动计划，新建和改扩建一批幼儿园。均衡发展和巩固提高义务教育，在基本解决“上学难”的基础上，着力化解“上好学难”的矛盾，促进教育公平，切实办好省级试点。优化发展高中阶段教育，适度调整普通高中和中职学校，力争新增1所省级示范学校、创建1所国家级示范学校。加快发展职业教育，支持随州职院申报与建设省级特色高职院校，启动职教园区和10个职教品牌建设，深化校企合作，提高办学质效。进一步完善助学贷款机制，确保家庭困难学生有书读。

（六）全面协调推进社会建设

实施就业援助行动。加大政策支持力度，增发小额担保贷款，扶持1000人成功创业，落实就业培训5000人次，加快市创业指导中心规范化、标准化建设，统筹解决好高校毕业生、农村转移劳动力、就业困难人员和退役军人等群体的就业问题。

完善社会保障体系。健全公共财政体系，创新财税征管方式，做到依法征收、应收尽收，增强对民生领域支出的保障能力。加强社会保险扩面征缴力度，逐步提高待遇水平。扎实推进城乡居民养老保险工作，进一步完善城乡最低生活保障、临时救助和农村五保供养制度。有序发展房地产市场，增加保障性住房和普通商品房的有效供给。拓宽住房公积金制度覆盖面，提高缴存水平，扩大贷款规模。加强价格管理，落实社会救助与物价上涨的联动机制，做好价格调节基金的征收和使用工作，确保物价总水平基本稳定。

统筹发展社会事业。巩固完善新农合制度、提高补贴标准，扩大国家基本药物制度的覆盖面，稳妥推进公立医院改革；加大医疗机构建设投入，实施一批乡镇卫生院和村卫生室的标准化改造，不断提高应对突发公共卫生事件的能力。进一步优化计生服务，坚决遏制违法生育，严格控制出生人口性别比。抓好国土资源管理，确保依法、依规、集约用地。严格环境准入制度，加强节能减排，保障环境安全。落实党的宗教政策，做好老干部工作，重视发展残疾人事业；充分发挥驻外机构、外事侨务、对台等部门对外交往和招商引资的桥梁纽带作用，统筹抓好统计、国调、审计、司法、档案、保密、史志、气象、水文、妇女儿童、慈善、红十字会、援疆和无偿献血工作；关心支持驻随部队建设，扎实做好国防教育、防空防震、民兵预备役、双拥共建和优抚工作。

全力维护社会稳定。加强和创新社会管理，进一步改进群众工作，畅通信访渠道，切实做好征地拆迁、企业改革、劳资纠纷、特殊人群和网络虚拟社会管理工作。健全人民调解、行政调解、司法调解“三大调解”的联动工作格局，统筹处理好各方面利益关系；加强法律援助，维护社会公平正义。支持工会、共青团等群团组织积极参与社会管理和公共服务。完善社会治安防控体系，增强人防、技防、物防能力，依法打击各类违法犯罪活动。健全公共安全体系，深入开展火灾等各类隐患的排查和整改行动，提高应急管理、处置公共安全事件的能力和水平。加强各类专业市场监管，切实保障食品药品安全；认真落实安全生产责任制，坚决遏制重特大事故发生，努力让人民群众更有安全感、归宿感和幸福感。

四、切实加强政府自身建设

“实干就是能力，落实就是水平。”新一届政府必须大力倡导省委省政府提出的“四文精神”，真抓实干、埋头苦干，全面加强自身建设，努力建设人民群众满意的政府。

（一）强化责任意识，提高行政执行力。坚持理论联系实际，从实践中来、到实践中去，不断提高政府及其公务人员谋划跨越和驾驭复杂局面的能力。坚持依法行政、诚实守信，既要依法依规，又要打破常规，言行必果、敢于担当。加强公务员队伍能力建设，健全责任体系，完善确责、履责、问责机制，使能力和职责相对称。扎实开展创先争优，深入推进治庸问责，着力解决不作为、慢作为、乱作为等问题。大力弘扬求真务实的作风，敢于直面困难，善于化解矛盾，全力抓好落实。

（二）强化效能意识，提高环境吸引力。深入推进行政管理体制改革，加强行政服务“三集中、三到位”工作，优化工作流程，创新服务方式，提高办事效率，清理规范所有行政收费项目，真正把随州建设成为全省发展成本最低、审批事项最少、服务效能最高的城市。提倡短文、短会、短话，切实改进文风会风，把更多的精力放在促发展、保稳定、惠民生上。

（三）强化服务意识，提高社会公信力。牢固树立执政为民、服务群众的理念，始终坚持重大决策依民而定、工作措施应民而出，在广泛征求群众意愿的基础上，市政府将集中财力，继续为市民办好十件实事：（1）扶持创业带动就业，增发小额担保贷款3000万元以上，城镇新增就业1.6万人，农村劳动力转移就业1.8万人。（2）推广试点经验，全面实施城乡居民养老保险，实现全覆盖。（3）实施文化惠民工程，规划建设季梁图书馆，完成农民体育健身工程50处以上。（4）启动新建1所城区义务教育学校，改扩建一批农村幼儿园。（5）启动市精神卫生中心、随县人民医院和中医院建设。（6）加快安居工程建设，新建公共租赁房3600套，解决757户中低收入群众的住房问题，增发货币化补贴1500户。（7）继续实施“千村万塘”工程，新建和改造堰塘2万口。（8）投资7850万元，解决14.5万农村居民和2万师生饮水难问题。（9）高标准完成800个20户以下自然村广播电视“村村通”建设任务。（10）为全市80岁以上的老人按月发放高龄补贴。

（四）强化廉政意识，提高制度约束力。自觉接受人大的法律监督、政协的民主监督，认真办理建议提案；广泛接受社会各界监督，虚心听取各民主党派、工商联、无党派人士的意见。认真落实党风廉政建设责任制，不断完善惩治和预防腐败体系。坚持用制度管权、管人、管物、管事，全面落实政务公开和政府信息公开制度，努力使行政权力在阳光下运行；不断完善招投标综合监管体系，构建公共资源交易平台；加强对重点领域、专项资金等方面的监管，突出抓好“十个全覆盖”工作，严肃查处损害人民群众利益的行为。

各位代表！形势催人奋进，使命神圣光荣，发展时不我待。让我们在中共随州市委的坚强领导下，在市人大、市政协的监督支持下，紧紧依靠全市人民，深入贯彻落实科学发展观，万众一心，扎实工作，以推动科学发展、跨越式发展的优异成绩，迎接党的十八大胜利召开！
